# Editorial for Brain Sciences Special Issue: “Advances in Skull Base Tumor Surgery: The Practical Pearls”

**DOI:** 10.3390/brainsci14040352

**Published:** 2024-04-01

**Authors:** Miguel Angel Lopez-Gonzalez

**Affiliations:** School of Medicine, Loma Linda University, Loma Linda, CA 92354, USA; mlopezgonzalez@llu.edu

The field of skull base surgery is unique; it involves the adequate and coordinated multidisciplinary interaction of multiple specialties, such as otorhinolaryngology, maxillofacial surgery, ophthalmology, neuro-anesthesiology, oncology, radiation oncology, neurophysiology, and neurosurgery. Young residents and fellows interested in this field need to learn and master the surgical skills required to tackle lesions located deep in the skull base with minimal morbidity.

The need to go back to the basics and learn and re-review human anatomy and classic skull base surgery approaches developed by the giants in our field cannot be overemphasized. The initial wave of skull base surgery started with approaches in sellar region developed by Schloffer in 1907 and subsequently modified by Hirsch and Cushing using the sublabial approach, and then followed by Dott, Guiot and Hardy, popularizing the transsphenoidal approach. The second wave in skull base surgery from 1980s was invigorated by the modern giants in skull base surgery, such as Yasargil, Hakuba, Al-Mefty, Dolenc, Spetzler, Kawase, Fukushima, Shekar, House, and Fisch, among others [1]. The third leap represents the endoscopic endonasal era that started in the 1990s, with the landmark paper by Jho and Carrau [2]. More knowledge has been accumulated, and now, skull base surgeons are able to access any skull base region either using a conventional lateral skull base approach, or endoscopic expanded endonasal techniques.

The trend of using minimal invasive approaches in skull base surgery is applauded, although it is critical to maintain a balanced judgement regarding when to use minimal invasive options versus the conventional skull base approaches. Current skull base surgeons need to have a good understanding of the reach of each of the approaches, profound anatomical knowledge developing via laboratory dissections, and surgical training. They also need to make an unbiased judgment on the appropriate procedure for each patient, with careful mental preparation and rehearsal to respond to all the possible intraoperative scenarios. The in-depth preoperative analysis of all the images (computed tomography, magnetic resonance, cerebral angiogram, etc.), as well as a consensus discussion with the other referred specialists when appropriate can provide an adequate and safe surgical plan. A perfectly orchestrated teamwork with surgeon leadership is mandatory. Closed and respectful communication with the entire surgical team before surgery is necessary to achieve the common goal of a successful operation. It is crucial to be versatile and provide exactly what each patient requires. There are new tools that can be utilized for preoperative planning and education, such as virtual, augmented, or mixed reality. When available, it is important to integrate these tools, although we need to keep in mind that the success of surgery comes with the adequate execution of the microneurosurgical skills, since technology, at this point, cannot perform the crucial delicate steps required for these formidable approaches.

In this Special Issue: “Advances in Skull Base Tumor Surgery: The Practical Pearls”, we integrate the experience of experts in the field, describing their techniques and pearls of wisdom to improve all the areas of skull base surgery from endoscopic endonasal to lateral skull base approaches, including videos for vestibular schwannoma and craniopharyngioma resection, as well as using the sphenoid ridge approach on pediatric patients. Among others, Liu and colleagues [3] present an elegant and detailed microsurgical anatomy review and surgical examples of jugular foramen tumors, ranging from using basic retrosigmoid approaches to combined retro and infralabyrinthine transjugular transcondylar high cervical approaches. Mukherjee and colleagues [4] present the reach of endonasal endoscopic skull base surgery for pathologies in the sellar–suprasellar area, orbital apex, and anterior cranial base in detail and transpterygoid approaches, with a thorough description of their practical pearls.

Overall, all the included articles encompass all the areas of skull base surgery. I recommend adding them with the surgical approach examples to the armamentarium of different modalities of treatment for skull base surgeons to deal with complex lesions in daily practice.
